# Ethnic and sex differences in the incidence of hospitalized acute myocardial infarction: British Columbia, Canada 1995-2002

**DOI:** 10.1186/1471-2261-10-38

**Published:** 2010-08-19

**Authors:** Aman PK Nijjar, Hong Wang, Hude Quan, Nadia A Khan

**Affiliations:** 1 Division of General Internal Medicine, University of British Columbia, BC, Canada; 2 Center for Health Evaluation and Outcomes Sciences, University of British Columbia, BC, Canada; 3 Department of Community Health Sciences, University of Calgary, AB, Canada

## Abstract

**Background:**

As populations in Western countries continue to change in their ethnic composition, there is a need for regular surveillance of diseases that have previously shown some health disparities. Earlier data have already demonstrated high rates of cardiovascular mortality among South Asians and relatively lower rates among people of Chinese descent. The aim of this study was to describe the differences in the incidence of hospitalized acute myocardial infarction (AMI) among the three largest ethnic groups in British Columbia (BC), Canada.

**Methods:**

Using hospital administrative data, we identified all patients with incident AMI in BC between April 1, 1995, and March 31, 2002. Census data from 2001 provided the denominator for the entire BC population. Ethnicity was determined using validated surname analysis and applied to the census and hospital administrative datasets. Direct age standardization was used to compare incidence rates.

**Results:**

A total of 34,848 AMI cases were identified. Among men, South Asians had the highest age standardized rate of AMI hospitalization at 4.97/1000 population/year, followed by Whites at 3.29, and then Chinese at 0.98. Young South Asian men, in particular, showed incidence rates that were double that of young Whites and ten times that of young Chinese men. South Asian women also had the highest age-standardized rate of AMI hospitalization at 2.35/1000 population/year, followed by White women (1.53) and Chinese women (0.49).

**Conclusions:**

South Asians continue to have a higher incidence of hospitalized AMI while incidence rates among Chinese remain low. Ethnic differences are most notable among younger men.

## Background

Coronary artery disease (CAD) is the leading cause of death in North America [1,2]. However, the majority of patients who suffer an acute myocardial infarction (AMI) will survive the initial event and continue to live with CAD [1]. The result of the tens of thousands individuals living with and managing CAD has an enormous social and financial impact on our health care systems. Research identifying populations that are at greater risk and in need of customized prevention strategies for CAD can potentially improve the health for a significant portion of the community.

While sex and age-standardized rates are often known for AMI admissions, the ethnic breakdown is more difficult to capture. The changing demographic of North America's ethnic groups calls for a closer look at AMI incidence among two rapidly growing minority ethnic groups, namely the South Asian and Chinese communities. It is already known that South Asians in Canada have a higher prevalence and mortality due to cardiovascular disease [3,4]. However, immigrants of Chinese origin demonstrate lower mortality rates from ischemic heart disease, although migrants who leave China have higher rates than their counterparts who remain in China [3,5,6]. Hypotheses to explain the varying rates of cardiovascular deaths in South Asians and Chinese include variation in AMI incidence. Other studies already completed in the UK demonstrate higher rates of AMI hospitalization among South Asian patients [7,8].

To better characterize the overall burden of CAD in different ethnic groups, we examined the incidence of hospitalization for AMI over seven years in British Columbia (BC), a multi-ethnic province of Canada. Age-standardized rates were determined for men and women of the three major ethnic groups of the province, specifically Chinese, South Asian, and Whites. Annual AMI incidence rates were also determined for each ethnic group for each individual year within the study period.

## Methods

We used a retrospective cohort study design and utilized routinely collected hospital administrative discharge data from all hospitals in BC (April 1, 1994 to March 31, 2003). All hospitals within the province are subject to mandatory reporting of discharge data. Index cases of AMI were identified using the International Classification of Diseases, 9^th ^Revision (ICD-9) and 10^th ^Revision (ICD-10) validated coding algorithms (410.x and I21.x, respectively) [9,10]. Ethnicity was determined using population registries that contain data on all registered persons within each province. Data from the different sources were linked via personal health numbers, a 10-digit unique identifier for health care services that is assigned to every Canadian resident and is present on population registries.

Index cases of AMI were limited to those occurring between April 1, 1995 and March 31, 2002. Patients were excluded if they were less than 35 years old, had a previous AMI diagnosis in the year prior to their admission, and a hospital stay less than one day. Excluding patients with stays of less than one day was instituted in order to avoid patients who were admitted for one-day cardiac procedures. Patients were also excluded if they were non-residents of the province. Previous work examining the accuracy of AMI coding algorithms in hospital discharge records in another Canadian setting (Ontario), has shown it to be reliable and have a high level of diagnostic agreement [11]. Both BC and Ontario share similar coding set-ups as laid out by the Canadian Institute of Health Information (CIHI). Direct comparisons of ICD-9 coding to clinical registry data for AMI identification demonstrated a sensitivity of 88.8%, specificity of 92.8%, and positive predictive value (PPV) of 88.5% [11].

Ethnicity information was not part of the hospital administrative data set for the AMI cohort. Instead, surname analysis allowed us to categorize patients into the subsets of Chinese (ancestry from China, Taiwan, or Hong Kong) or South Asian (ancestry from India, Pakistan, or Bangladesh). Surname analysis was conducted by merging provincial population registries with Quan's Chinese name list [12] and the Nam Pehchan computer program [13] to define Chinese and South Asian ethnicity, respectively. Compared to self-report, the sensitivity for Quan's surname algorithm was 78% and specificity was 99.7% [12]. Nam Pehchan's program has been previously validated and shown to recognize South Asian surnames with a sensitivity of 90-94% and specificity of 99.6% [14,15]. While misclassification can still occur, for example in the instance of changing surnames following an interracial marriage, Quan's algorithm found only a small drop in sensitivity when comparing married females (Sens = 73.2%) to never-married females (Sens = 76.7%) [12]. During a validation of the Nam Pehchan program, only 0.05% of the 356,555 names examined were found to have mixed components of South Asian and non-South Asian origins [14]. Within our study, remaining patients who were non-South Asian, non-Chinese were categorized as "White." Since Chinese and South Asians make up the bulk of the minority population in BC, the number of other minority groups that were classified as "White" remained relatively small. Persons considered part of the visible minority that are neither Chinese nor South Asian, represent only 6.67% of BC's entire population [16].

Population data was extracted through the Canada 2001 census [16]. The entire population of BC was used as the reference population for direct age standardization, categorized by sex and divided into age groups of ten-year increments. Furthermore, census data allowed us to determine the age breakdown of our three ethnic groups, namely Chinese, South Asian, and White. These data were divided into the same age strata as the reference population (BC population).

New AMI cases over the seven year study period were stratified by sex and the same age group categories. Surname analysis was used to determine the ethnicity of the AMI cases. Within each age stratum for each ethnic group, the specific incidence rate of AMI hospitalization was calculated, with corresponding 95% confidence intervals. Age-specific rates were then multiplied to the corresponding age strata of the reference population.

AMI rates for each individual year in the seven-year study period, stratified by ethnicity, were also determined to explore time trends. Confidence intervals were calculated for the annual crude rates. Sex-specific analysis was not conducted due to a reduced sample size following the year by year breakdown.

Calculations were made using Microsoft Excel while confidence intervals were determined by SAS statistical software version 9.2 (SAS Institute Inc, Cary, North Carolina). Approval for the study was obtained through the British Columbia Providence Health Care ethics board.

## Results

Over the seven year study period (1995-2002), there were a total of 34,848 new cases or 'incident' AMI hospitalizations detected. The population of BC, age 35 and over, in 2001, was estimated at 2,168,715 for an overall AMI rate per 1000 persons per year of 2.30. The corresponding Chinese population of BC numbered 192,155 (8.86%). The South Asian population comprised of 87,965 persons (4.06%). Of the total number of AMIs, Chinese patients overall had 820 (2.35%) while South Asians had 1944 (5.58%).

### AMI Incidence Among Men

Men made up 23,205 (66.6%) of all AMI cases. AMI incidence rates for men are shown in Table 1. Overall, male South Asian patients showed a higher incidence of AMI in all age groups compared to Chinese and Whites and the highest age-standardized rate. South Asian men also demonstrated high rates of AMI at earlier ages than the other two ethnic groups. A large increase in incidence occurred among South Asian men aged 45-54, where incidence rates (3.44/1000/year) were almost double the rate of White men in the same age group. White men do not show a similar increase until the ages of 55-64. The age-specific incidence per 1000 South Asians per year in the two youngest age groups were approximately double that of the majority White population. In the older age groups, the incidence rate of South Asian patients was about 1.5 times that of Whites. Chinese male patients in all age groups were at least three times less likely than Whites to suffer an AMI. In the youngest age group of 35-44 years, Chinese were at least six times less likely to have an AMI, compared to Whites.

**Table 1:** Incidence Rates and Number of AMI Hospitalizations in Men, by ethnicity (1995-2002)

	Chinese			South Asian			White		
	**Total Cases**	**Population**	**Age-specific incidence/1000/year (95% CI)**	**Total Cases**	**Population**	**Age-specific incidence/1000/year (95% CI)**	**Total Cases**	**Population**	**Age-specific incidence/1000/year (95% CI)**

35-44 years	15	30100	0.07 (0.04-0.11)	96	15445	0.89 (0.71-1.07)	927	274845	0.48 (0.45-0.51)

45-54 years	84	28990	0.41 (0.33-0.50)	287	11930	3.44 (3.04-3.83)	3175	256110	1.77 (1.71-1.83)

55-64 years	97	12770	1.09 (0.87-1.30)	408	8790	6.63 (6.00-7.26)	4751	167350	4.06 (3.94-4.17)

> 65 years	349	17490	2.85 (2.55-3.15)	592	7625	11.09 (10.23-11.95)	12424	211565	8.39 (8.25-8.53)

Crude Rate/1000/year			0.87			4.51			3.34

**Age Standardized Rate/1000/year**			**0.98**			**4.97**			**3.29**

### AMI Incidence Among Women

Data on AMI incidence in women is shown in Table 2. South Asian women showed the highest age-standardized rates at 2.35 per 1000 per year, followed by White and then Chinese women.

**Table 2:** Incidence Rates and Number of AMI Hospitalizations in Women, by ethnicity (1995-2002)

	Chinese			South Asian			White		
	**Total Cases**	**Population**	**Age-specific incidence/1000/year (95% CI)**	**Total Cases**	**Population**	**Age-specific incidence/1000/year (95% CI)**	**Total Cases**	**Population**	**Age-specific incidence/1000/year (95% CI)**

35-44 years	<5	36525	0.004 (0-0.012)	8	15360	0.07 (0.02-0.13)	188	283900	0.09 (0.08-0.11)

45-54 years	15	31560	0.07 (0.03-0.10)	31	11900	0.37 (0.24-0.50)	718	259220	0.40 (0.37-0.42)

55-64 years	32	14430	0.32 (0.21-0.43)	109	8875	1.75 (1.43-2.08)	1407	167535	1.20 (1.14-1.26)

> 65 years	227	20290	1.60 (1.39-1.81)	413	8040	7.34 (6.65-8.03)	8494	268080	4.53 (4.43-4.62)

Crude Rate/1000/year			0.38			1.81			1.58

**Age Standardized Rate/1000/year**			**0.49**			**2.35**			**1.53**

Age-specific AMI incidence rates remained relatively similar between South Asian and White women up until the 55-64 age group. From the 45-54 group, White women showed an increase of three times to the rate of those aged 55-64. In that same increment change, South Asian womens' rate jumped almost five times. Among women in the two oldest age groups (55-64 years and >65 years), South Asians had the highest incidence, with event rates of 1.75 per 1000 persons per year among women aged 55-64 and 7.34 among those aged 65 and greater. Overall, the age-standardized rate of South Asian women was 1.5 times higher than that of White women.

Chinese women had lower incidence rates in all categories compared to Whites. Their age-standardized rate was three times lower than White women. In the youngest age group, Whites had incidence rates that were nine times higher than those of Chinese. In women aged 65 and greater, the rate was 2.8 times higher in White women compared to Chinese.

### Annual Trends

**Figure 1:**
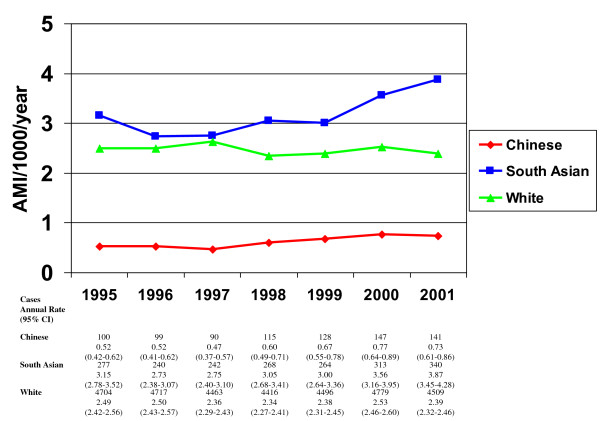
**Crude annual AMI rates**. White--green triangle. Chinese--red diamond. South Asian--blue square

Total number of cases, crude AMI rates and 95% confidence intervals by each year are shown in Figure 1. Each ethnic group represents both men and women. Overall, there was little change in the annual rates of AMI for Chinese and Whites. South Asians exhibited higher rates than Whites for the majority of the study period, with South Asians showing an increase after 1999.

## Discussion

We found that South Asians had considerably higher rates of hospitalization for AMI compared to Whites. Across all ages, people of Chinese descent had significantly lower rates of AMI hospitalization in comparison to the two other ethnic groups.

Several studies reported South Asian immigrants have a significant increase in CAD compared to Whites [17]. However, most of these studies describe prevalence of cardiac disease or mortality. There is considerably less data focused on incidence in South Asian patients when they immigrate to Western countries. In two earlier incidence studies in Scotland and Sweden, South Asians patients were found to have a higher incidence of AMI compared to White patients [18,19]. These studies reported an age-standardized risk increase of 1.5-2.0 compared to White patients, consistent with our findings. In the sex stratified analyses of these two studies, South Asian women demonstrated a two-fold increase in risk of AMI compared to their non-South Asian female population. In our study, South Asian women had an AMI rate 1.5 times that of White women. However, the Scottish study also included out-of-hospital deaths and other international studies found that the incidence of coronary events in Glasgow, Scotland, were among the highest in the world [20].

AMI incidence in Chinese immigrant populations is less well known. In China, AMI incidence is quite variable with age-adjusted rates ranging from 3.3 to 108.7 per 100,000 in men 1.0 to 40.0 per 100,000 in women [21]. However, these data are over 15 years old and there have been significant changes in detection of AMI, health care access, and the occurrence of AMI given the rapid increases in diabetes, smoking, and obesity in China over the past two decades [22-24]. Our findings are more consistent with rates reported among people of Chinese descent living in Singapore. Furthermore, in Singapore, South Asians were also found to have significantly higher rates of AMI than their Chinese counterparts [5].

At the Canadian national level, overall rates for AMI hospitalization have also been recently described. BC rates were similar to the published national crude rate [25] and consistent with province-specific data presented from the Canadian Institute for Health Information [26]. Similarly, the overall provincial rate of AMI hospitalization in BC is close to national rates in the United States [27] but lower than rates in the United Kingdom [28]. However, it is important to note that in a region with overall lower rates of AMI hospitalization, there is still a wide variation observed in our different ethnic groups.

Another contributor to the incidence rates described in our study is that the definition of myocardial infarction underwent significant changes by the year 2000 with the introduction of highly sensitive and cardiac-specific troponin assays [29]. The use of troponin testing in patients with acute coronary syndrome resulted in a consensus change in the definition of AMI, with an emphasis on troponins as the new gold standard. With the augmented sensitivity of the assay, this likely had an effect on increased detection of AMI and may explain, in part, the rise in AMI rates after 1999, as shown in Figure 1. Why this had a greater impact on South Asian rates in our study is not clear. However, it may simply reflect population changes. Although we used 2001 census data, there was a 37.7% growth rate in the South Asian population in Canada from 2001 to 2006 [30]. The rise in AMIs among South Asians may be a result of a larger at-risk population that began to increase in 2000. While the rise and higher rate of AMIs among South Asian patients is considerable, it still accounts for a small number of the overall number of AMIs and therefore the calculations are potentially unstable, as evidenced by the wider confidence intervals.

In age-specific analysis, young South Asian men in particular carried a large burden of AMI when compared to our other populations. This is consistent with previous prevalence studies [31]. The 45-54 age group demonstrates the largest differential in AMI hospitalization rates between Whites and South Asians. In this younger group, there is an almost two-fold increase in AMI hospitalization among South Asians, compared to Whites. The increased risk may be partially explained by higher levels of diabetes, dyslipidemia, and intraabdominal adiposity among young South Asians. Specifically, a high apolipoprotein B(100)/apolipoprotein A-1 ratio was shown to be more common among native South Asians with AMI than compared to individuals from other countries [31]. Other reasons to explain the overall increased incidence include an overall high prevalence of metabolic syndrome in the South Asian population. South Asians have been shown to have a high percentage of body fat, abdominal obesity, insulin resistance, hyperinsulinemia, and low muscle mass [32]. Individuals with the elements of metabolic syndrome face a 2-3 fold increased risk of cardiovascular mortality [33]. Some researchers also propose that South Asians may have an exaggerated response to several traditional cardiovascular risk factors. In particular, South Asians who have migrated to Western countries may experience additional psychosocial and metabolic stresses that contribute to an accelerated progression of cardiovascular disease [34].

Among South Asian women, AMI incidence was similar to their White counterparts, until the post-menopausal period where South Asian women show a 1.5 times increase compared to White women. It is already known that coronary artery disease is less prevalent among pre-menopausal women. In general, prevalence rates for both men and women do not approach parity until patients reach their sixties [35]. Any protective effect of the pre-menopausal period is significantly attenuated after the age of 65 years, exposing South Asian women to the same high levels of cardiac risk factors that are present in the overall South Asian community.

Both Chinese men and women demonstrated low rates of AMI. Explanations for the low rate of coronary heart disease among individuals of Chinese descent focus on an overall lower rate of traditional risk factors. Some studies highlight the dietary pattern of low saturated fat and cholesterol intake [36]. Compared with Western populations, the average level of total cholesterol and prevalence of dyslipidemia is relatively low [37]. Even in patients with coronary heart disease, small comparative studies between Chinese and European patients demonstrate that Chinese patients have significantly lower rates of hypertension, dyslipidemia, and abnormal glucose metabolism [38]. Most recently, the INTERHEART China study showed that Chinese participants had lower levels of lipids, diabetes, psychosocial factors, and BMI/waist to hip ratios. Furthermore, the strength of association of waist to hip ratio and AMI was lower in China than other countries. Overall, the nine modifiable risk factors of INTERHEART accounted for 89.4% of AMI risk [39].

Limitations of this observational study include misclassification of ethnicity. However, this misclassification is likely to be non-differential and would tend to underestimate any differences between the ethnic groups. In addition, we are not able to capture out of hospital AMI, patients who were misdiagnosed, or patients who died from AMI at home or on the first day of hospital admission. While we are unable to determine if ethnic differences exist in the occurrence of out-of-hospital AMI, there is recent work suggesting no difference in mortality due to out of hospital cardiac arrest between White and South Asian patients [40].

## Conclusions

The widely varying rates of incident hospitalization for AMI present an opportunity for further research, intervention, and program planning. Determining the protective factors that keep AMI incidence low among Chinese is essential to prevent the community from developing the rates of their White and South Asian neighbors. Targeting young South Asians and investigating the reasons for an early onset and high level of risk factors may prevent future complications such as AMI. From our findings, it may be necessary to begin earlier screening for cardiovascular risk factor and aggressive risk factor reduction in the South Asian population.

Although women did not demonstrate as wide of a difference between ethnic groups, post-menopausal South Asian women still represent a high risk group. Programs and policies aimed at reducing the CAD burden in the South Asian population require attention to both men and women. Finally, as immigration from southern Asia and China will only increase, we need to further tailor our prevention strategies and periodically evaluate whether they are effective for the entire population.

## Abbreviations

AMI: acute myocardial infarction; CAD: coronary artery disease; BC: British Columbia; ICD: International Classification of Diseases

## Competing interests

The authors declare that they have no competing interests.

## Authors' contributions

APKN drafted the original manuscript. NAK and HQ participated in the original design of the study. HW ran all the statistical analyses. NAK participated in manuscript revisions. All authors read and approved the final manuscript.

## Pre-publication history

The pre-publication history for this paper can be accessed here:

http://www.biomedcentral.com/1471-2261/10/38/prepub
